# Pelvic Abscess

**Published:** 1846-04

**Authors:** 


*Pelvic Abscess.*—Dr. Henry Bennett describes a case of pelvic abscess, following phlegmonous inflammation of the cellular tissue of the lateral ligaments, which burst into the vagina. By rest in bed, emollient injections, tepid hip-baths, and salines, the immediate effects of the inflammation were removed, and the remaining tumour occupying the seat of the abscess, was gradually absorbed. A large ulceration of the cervix uteri was also cured by repeated cauterisations with the nitrate of mercury and the nitrate of silver.—*Ibid.*

